# Treatment and Care of Patients with ST-Segment Elevation Myocardial Infarction—What Challenges Remain after Three Decades of Primary Percutaneous Coronary Intervention?

**DOI:** 10.3390/jcm13102923

**Published:** 2024-05-15

**Authors:** Vittorio Zuccarelli, Stefano Andreaggi, Jason L. Walsh, Rafail A. Kotronias, Miao Chu, Jonathan Vibhishanan, Adrian P. Banning, Giovanni Luigi De Maria

**Affiliations:** 1 Oxford Heart Centre, Oxford University Hospitals NHS Trust, Oxford OX3 9DU, UK; vittorio.zuccarelli@cardiov.ox.ac.uk (V.Z.); stefano.andreaggi@studenti.univr.it (S.A.); jason.walsh@queens.ox.ac.uk (J.L.W.); rafail.kotronias@cardiov.ox.ac.uk (R.A.K.); miao.chu@cardiov.ox.ac.uk (M.C.); jonathan.vibhishanan@ouh.nhs.uk (J.V.); adrian.banning@ouh.nhs.uk (A.P.B.); 2 Division of Cardiology, Department of Medicine, University of Verona, 37129 Verona, Italy; 3 Division of Cardiovascular Medicine, Radcliffe Department of Medicine, University of Oxford, Oxford OX1 2JD, UK; 4 National Institute for Health Research (NIHR), Oxford Biomedical Research Centre, Oxford OX3 9DU, UK

**Keywords:** ST-elevation myocardial infarction, primary percutaneous coronary intervention, coronary no-reflow

## Abstract

Primary percutaneous coronary intervention (pPCI) has revolutionized the prognosis of ST-segment elevation myocardial infarction (STEMI) and is the gold standard treatment. As a result of its success, the number of pPCI centres has expanded worldwide. Despite decades of advancements, clinical outcomes in STEMI patients have plateaued. Out-of-hospital cardiac arrest and cardiogenic shock remain a major cause of high in-hospital mortality, whilst the growing burden of heart failure in long-term STEMI survivors presents a growing problem. Many elements aiming to optimize STEMI treatment are still subject to debate or lack sufficient evidence. This review provides an overview of the most contentious current issues in pPCI in STEMI patients, with an emphasis on unresolved questions and persistent challenges.

## 1. Introduction

ST-segment elevation myocardial infarction (STEMI) occurs when an atherosclerotic plaque ruptures or erodes, triggering the coagulation cascade and platelet activation, resulting in intraluminal thrombus formation and coronary artery occlusion, leading to myocardial necrosis. STEMI constitutes a life-threatening medical emergency due to the imminent risk of arrhythmic events, mechanical complications, and an acute and long-term risk of heart failure.

In 1977, Dr Grüntzig performed the first successful percutaneous transluminal coronary angioplasty (PTCA) in stable coronary artery disease, demonstrating the feasibility of catheter-based revascularization [1]. In 1983, Dr. Hartzler introduced primary angioplasty in the treatment of acute myocardial infarction (MI) [2]. Despite initial enthusiasm, early trials investigating balloon angioplasties following thrombolytic therapy did not demonstrate superiority over thrombolytic therapy alone [3,4]. However, primary PTCA alone was subsequently shown to be superior to thrombolytic therapy alone in terms of mortality, re-infarction, stroke, and major bleeding [5,6]. Angioplasty faced three main issues at the time: intravascular thrombosis, vascular occlusion due to elastic recoil, and neointimal proliferation leading to restenosis [7,8].

In 1991, these challenges were addressed with the introduction of bare-metal stents (BMSs), providing a metal scaffold aiming to reduce restenosis and re-occlusion following PTCA [9]. BMS demonstrated its superiority over balloon angioplasty alone [10,11]. Subsequently, several trials including STENT-PAMI, FRESCO, and CADILLAC established coronary stenting as the standard of care reperfusion strategy for MI to reduce restenosis, re-occlusion, and recurrent ischemia [12,13,14]. Despite the excitement surrounding BMSs, intimal hyperplasia leading to in-stent restenosis remained a significant issue. In the early 2000s, the first drug-eluting stents (DESs) were introduced. Following this, several studies confirmed the safety and efficacy of pPCI using BMSs and DESs in STEMI [15,16,17]. Over the past decade, DES technology has continued to improve with better platforms and antiproliferative drugs leading to lower rates of restenosis [18,19,20]. Over the years, several new stent innovations have failed to show efficacy, such as bioresorbable scaffolds [21,22].

As a result of these advancements, pPCI became established as the gold standard strategy for treating STEMI compared to thrombolysis, leading to a proliferation of catheterization laboratories around the world. Another major step forward has been the shift from femoral to radial access, reducing vascular and bleeding complications [23,24].

STEMI represents one third of all admissions for acute coronary syndromes [25]. The global prevalence of STEMI is estimated at 4% in individuals <60 years old and 9.5% in individuals >60 years old [26]. In the past thirty years, STEMI presentations have declined in incidence in high-income nations [27], largely due to an increased awareness of cardiovascular risk factors and widespread primary prevention strategies. Specifically, in the US, between 1997 and 2005, the incidence rate of STEMI decreased from 121 to 77 per 100.000 people [28].

Additionally, data from a wide national registry confirm a significant decrease in 6-month mortality following acute MI for all patients with acute coronary syndromes between 1995 and 2015. Although mortality rates continued to decline in STEMI patients until 2015, mortality in patients with NSTEMI has remained stable since 2010 [29].

Despite improvements in STEMI care, no overall improvement in mortality was seen in the first two decades of this century. However, once adjusting for comorbidities and high-risk features such as cardiac arrest on presentation, there was a significant decrease in risk-adjusted mortality. This demonstrates that patients presenting to the catheterization laboratory have become sicker over time [28]. Data coming from European registries established that in high-income European countries, 1-year mortality in pPCI-treated patients substantially decreased from 2003 to 2018, with the largest absolute mortality decline occurring in the first 30 days. The pooled in-hospital mortality rate for patients with ST-elevation myocardial infarction ranges between 4% and 12%. Additionally, mortality at the one-year follow-up is estimated to be around 10% [30,31]. A recent meta-analysis, which included 91 cohort studies involving around 200,000 patients, identified advanced age, out-of-hospital cardiac arrest, cardiogenic shock, delay in symptoms-to-balloon, and door-to-balloon time as the main predictors of death [32].

There is little doubt that a major contributor to this steep decline in mortality comes from the prompt mechanical restoration of the infarct-related artery (IRA) via primary percutaneous coronary intervention (pPCI). In 1993, the PAMI trial first demonstrated the superiority of pPCI over thrombolysis, with lower in-hospital and 6-month rates of death and re-infarction, as well as a reduced incidence of intracranial bleeding [33].

Despite this progress, challenges persist in STEMI care, some of which have not been fully addressed. At a broad level, improving overall in-hospital mortality remains a major challenge. Further, the growing burden of heart failure in STEMI survivors is a growing challenge, which significantly impacts long-term survival and quality of life, placing strain on healthcare systems [34].

**Figure 1:**
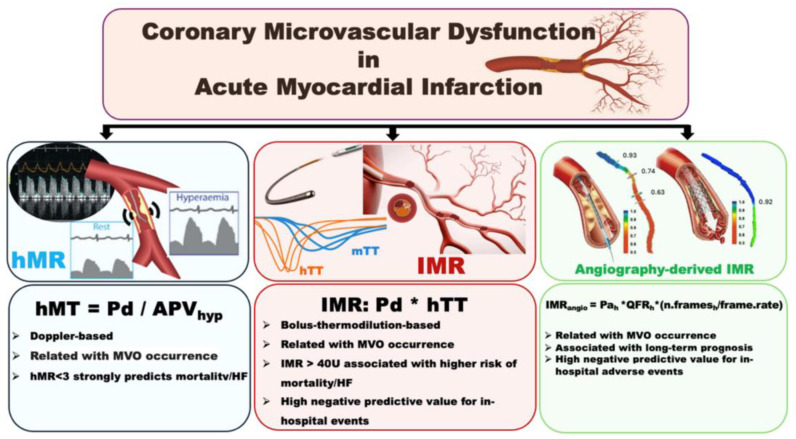
Coronary microvascular dysfunction in acute myocardial infarction. Abbreviations: hMR = hyperaemic microvascular resistance; hMT = hyperaemic mean transit time; Pd = mean distal coronary pressure; APVhyp = hyperaemic average peak flow velocity; IMR = index of microcirculatory resistance; hTT = hyperaemic transit time; MVO = microvascular obstruction; HF = heart failure; IMRangio = angiography-derived index of microcirculatory resistance; Pah = hyperaemic aortic pressure; QFR = quantitative flow ratio; n.frames_h_ = number of frames acquired during hyperaemia.

This review seeks to identify and explore specific challenges in optimising STEMI care and delineate new avenues of investigation in STEMI care in the years ahead (Figure 1).

## 2. Ischaemic Time: Can We Make It Shorter?

The extent of myocardial ischemia is one of the main determinants of infarct size, as clearly demonstrated in both experimental models [35] and clinical practice [36,37].

The concept of door-to-balloon time (DTB), defined as the time-interval between arrival to hospital and coronary flow restoration via pPCI, has emerged as a key quality indicator of performance for ambulance networks and hospitals. However, a national study in the United States showed no improvement in mortality despite an average reduction in DTB from 83 to 67 min [38].

A subsequent analysis on the same registry found the same association in the population as a whole; however, in patient-specific groups, reduced DTB time was associated with improved short-term mortality [39].

Symptom-to-balloon (STB) has since emerged as a more reliable mortality predictor [40,41]. The literature has reported a clear relationship between mortality and time delay from symptom onset to fibrinolysis [42] and pPCI [43] in STEMI patients. Observational studies have shown that most of the time delay in STB is represented by the time interval between symptom onset and hospital arrival, with factors such as advanced age, diabetes, and initial admission to a non-PCI centre identified as the main predictors of prolonged STB time [44]. Interestingly, the SARS-CoV-2 pandemic demonstrated the impact of treatment delay on STEMI outcomes in the present era [45].

In response, efforts have aimed to reduce prehospital admission delays in STEMI. Pre-hospital ECG diagnosis and direct transfer to the catheterization laboratory have resulted in a significantly shorter STB time [46]. Equally, the value of earlier symptom recognition and the seeking of medical attention (e.g., pain to first medical contact) have been recognized as important areas to invest. Despite efforts to promote early symptom recognition through media campaigns, certain populations still exhibit reluctance to seek prompt medical assessment for heart attack symptoms, leading to poorer outcomes [47,48]. Sociodemographic factors, such as lower education levels, advanced age, and rural residency, contribute to delays in seeking medical attention. This suggests that policymakers should target these populations by implementing tailored educational programmes and enhancing accessibility to healthcare services in these groups, aiming to mitigate delays in seeking care.

Notably, the financial impact associated with myocardial infarction is high, as a result of the increasing sophistication of acute care, hospital stay in intensive care environments, frequent readmissions, and long-term comorbidities [49]. Consequently, considerable effort and resources are now essential in primary prevention to raise awareness in the public and to further improve pPCI networks. This is a major challenge as national healthcare services, acute and emergency departments, and ambulance services are already over-stretched and working at maximum capacity and at the limits of financial sustainability.

## 3. Primary PCI in STEMI Patients Complicated by Cardiogenic Shock

The incidence of cardiogenic shock in patients admitted with myocardial infarction ranges between 3 and 13% [50]. Despite a widespread implementation of early revascularization with subsequent mortality reduction, cardiogenic shock remains the leading cause of death in STEMI patients [51,52]. Data from randomized controlled trials (RCTs) such as the SHOCK-trial, IABP-Shock II, and CULPRIT-Shock, highlight that mortality between 30 days and 1 year in these patients ranges from 6.7% to 12% [51,52,53,54].

RCTs have proven that an invasive approach with early revascularization in STEMI patients with cardiogenic shock reduces the overall and cardiovascular mortality at long-term follow-up [55]. Real-world data from multicentre registries have equally confirmed the benefit of early invasive management along with the reduction in time between first medical contact and pPCI [55,56,57].

Most STEMI patients with cardiogenic shock have multivessel coronary artery disease (CAD). Multivessel CAD constitutes an independent predictor of in-hospital mortality [58]. How and to what extent to revascularize STEMI patients with multivessel CAD and cardiogenic shock are still a matter of debate. Although achieving a complete coronary revascularization improves myocardial perfusion and cardiac output, multivessel PCI is strongly related to increased procedural time and higher rates of procedural complications, which have a magnified impact on a patient in cardiogenic shock [59].

The CULPRIT-Shock trial demonstrated that the early revascularization of the IRA alone at the index procedure, followed by the staged completion of revascularization, improved 30-day survival rates and reduced the need for renal replacement therapy compared to immediate multivessel PCI at the index procedure [51]. However, long-term outcomes showed no significant difference in mortality between the two strategies at one year [60]. Interestingly, the IRA-only PCI group had higher rates of heart failure rehospitalisation and repeat revascularization at one year [61]. The trial experienced a notable rate of crossover between groups, indicating the difficulty in achieving complete revascularization, particularly in patients with chronic total occlusion [61].

Data from real-world registries have started to highlight that STEMI patients complicated by cardiogenic shock can benefit from complete revascularization in terms of all-cause death and non-IRA repeat revascularization both at short- and long-term follow-up [62,63]. These results underscore the fragmented and contrasting evidence on the topic, calling for additional RCTs to help better define patient selection criteria, optimal approaches, and timing for complete revascularization in STEMI complicated by cardiogenic shock. Additionally, the impact of functionally significant non-culprit vessel disease on the prognosis of cardiogenic shock after myocardial infarction remains to be investigated, as does the impact of the occurrence and extent of microvascular obstruction in this category of patients.

A further matter of debate relates to the role of temporary mechanical circulatory support (MCS).

The IABP-SHOCK II trial has confirmed no significant prognostic benefits associated with the use of an intra-aortic balloon pump (IABP) in STEMI patients in cardiogenic shock undergoing pPCI [52,53,54,55,56,57,58,59,60,61,62,63,64]. However, significant variability in the timing of IABP insertion and revascularization protocols with significant crossover rates (ca 10%) between groups should be noted.

The early implantation of a micro-axial pump (Impella CP) as mechanical circulatory support is independently associated with improved survival, in the context of shock before the initiation of inotropes or vasopressors and without delaying reperfusion [65]. Furthermore, survival has been reported to decline in proportion to time delay between shock onset and MCS initiation, decreasing from 65% when MCS is implemented <1.25 h to 26% when MCS is started after 4.25 h [65]. Despite demonstrating a superior hemodynamic profile compared to IABP, data from RCTs have failed to show differences in short- and long-term mortality between IABP and Impella in patients with STEMI and cardiogenic shock [66]. Moreover, the use of Impella has been associated with a higher rate of bleeding complications.

Encouraging data emerged from the most recent RCT, the DanGer Shock trial, involving patients with STEMI complicated by CS randomized to receive a microaxial flow pump (Impella CP) plus standard care or standard care alone. The use of Impella CP led to a lower risk of death from any cause at 3 months even though patients in the Impella arm experienced a significantly higher rate of device-related complications, in terms of major bleeding, limb-ischaemia, haemolysis, device failure or worsening aortic regurgitation, and a higher rate of renal replacement therapy [67].

Which MCS for which STEMI patient remains unanswered. The SCAI-shock (Society for Cardiovascular Angiography & Interventions) classification, the phenotype of cardiac failure, and vascular access anatomy all certainly play a role in choosing the most appropriate modality of hemodynamic support. Further, the timing of MCS insertion has been recognized as an independent predictor of prognosis. Promising data, despite coming from observational studies, reported that the combination of Impella-supported pPCI and of complete revascularization in STEMI patients in cardiogenic shock translated into a reduction in short-term mortality, in particular in those patients in whom Impella was started before pPCI [68].

Further evidence needs to be built to clarify the role of MCS in this clinical scenario. Ongoing trials, such as the ANCHOR trial (NCT04184635) and RECOVER IV (NCT05506449), will help to shed more light on this highly debated topic.

## 4. Anticoagulant Treatment in the Acute Phase—When Should It Be Started?

Anticoagulation plays a key role in the initial treatment of STEMI patients managed with an invasive strategy. Unfractionated heparin has been established as the standard of care in patients with STEMI undergoing pPCI due to its favourable risk/benefit profile. However, there is a notable absence of high-quality evidence regarding pre-treatment with UFH, defined as the intravenous administration of UFH at 70–100 units/kg once the diagnosis of STEMI has been confirmed and before arrival in the catheterization laboratory, in patients with STEMI undergoing primary PCI. This is reflected in current European–American guidelines where UFH is endorsed for use during PCI, but there are no clear recommendations for UFH pre-treatment prior to arrival in the catheterization laboratory.

In the literature, only observational studies and non-randomized clinical trials have dealt with this topic [69,70]. At present, there is no conclusive evidence on absolute risk difference between anticoagulation pre-treatment and anticoagulation during pPCI. Much of the evidence supporting the potential benefits of UFH pre-treatment has been derived from trials involving medically managed patients with unstable angina or non-ST-segment elevation MI. Heparin pre-treatment has been related to a lower risk of intracoronary thrombus, a lower rate of total vessel occlusion, and enhanced reperfusion success with a significant impact on 1-year survival [71,72], without a significant increase in the rate of major bleeding [71]. The ongoing trial UFH-STEMI, which compares final TIMI flow in patients randomized 1:1 to early UFH administration at first medical contact versus administration after coronary angiography, will shed new light on this topic.

Alternatively, enoxaparin and bivalirudin could be considered in patients with STEMI undergoing pPCI. Enoxaparin has been demonstrated to be superior to UFH in decreasing clinical ischaemic outcomes such as CV death, MI complications, and PCI failure, without significant differences in major and minor bleeding [72]. Recently, an RCT including East-Asian patients has proven that, in STEMIs undergoing primary PCI, anticoagulation with bivalirudin, followed by a post-PCI high-dose infusion for 2–4 h, significantly reduced the composite endpoint of all-cause mortality and major bleeding at 30 days, compared with UFH [73].

## 5. Antiplatelet Therapy Timing in Primary PCI: What and When?

Antiplatelet agents are the cornerstone in the acute treatment of STEMI, and the choice of antiplatelet regimen should take both the patient’s thrombotic and bleeding risk profile into account. In addition to aspirin, a potent P2Y12 receptor inhibitor (prasugrel or ticagrelor) is recommended as the default strategy for patients with acute coronary syndrome [74,75]. According to European and American Guidelines, clopidogrel should only be used when prasugrel and ticagrelor are contraindicated, or as an alternative when bleeding risk is high, such as in the elderly or after fibrinolysis [76,77].

Debate persists on the optimal timing for loading doses of P2Y12 receptor inhibitors. The ATLANTIC trial compared ticagrelor administration before coronary angiography during ambulance transfer versus in-hospital before angiography for STEMI patients. The trial found no significant improvement in primary endpoints, such as ST-segment elevation resolution or TIMI flow, with pre-treatment [78]. Further, real-world multicentre registry data support the lack of benefit in terms of hard cardiovascular outcomes like all-cause mortality, myocardial infarction, or stent thrombosis at 30 days [79].

In NSTEMI, the ACCOAST trial showed that prasugrel pre-treatment did not reduce the incidence of thrombotic complications in either the overall population or among patients undergoing PCI, with a lack of protection against ischemic events consistently shown across all pre-specified subgroups, including diabetic and elderly patients [80]. Additionally, the COMPARE-CRUSH trial investigated the impact of pre-treatment with crushed versus whole prasugrel prior to pPCI. No notable difference in TIMI-3 initial flow or complete ST-segment resolution one hour after pPCI was evident between groups [81]. Comparable results were confirmed at 1-year follow-up with no difference in terms of clinical outcomes [82]. However, a sub-analysis of this trial highlighted that the pre-hospital administration of crushed prasugrel significantly improved postprocedural TIMI 3 flow rates in STEMI patients with an ECG-based large myocardial area at risk [83]. Hence, these findings may be hypothesis-generating in support of the use of prasugrel pre-treatment in high-risk ST-elevation myocardial infarction (STEMI) patients undergoing primary percutaneous coronary intervention (pPCI).

In conclusion, in the STEMI clinical setting, the higher degree of thrombotic burden in the IRA could lead to speculation about a higher benefit from an early administration of a potent P2Y12 inhibitor, but evidence in this regard is missing.

## 6. Intravenous/Intracoronary Antiplatelet Agents: Do They Still Have a Role?

Peri-interventional intravenous/intracoronary antiplatelet agents typically include glycoprotein IIb/IIIa inhibitors (GPI). Most of the trials evaluating GPI in PCI-treated patients pre-dated the routine use of last-generation P2Y12 inhibitors and most of these trials included mainly NSTEMI patients [84,85,86]. Data from initial studies showed a clinical benefit of routine GPI administration in the setting of primary PCI [87,88].

However, more recent data do not support the routine use of GPI. The On-TIME 2 trial randomized patients to a pre-hospital administration of tirofiban or placebo after a loading dose of clopidogrel, showing no additional significant survival benefit nor reduction in re-infarction at 1 year, despite a lower rate of stent thrombosis in the active arm [89]. Similarly, the BRAVE-3 trial showed that routine abciximab administration after a loading dose of clopidogrel did not achieve a reduction in infarct size nor improved survival rate at 30 days, compared to placebo [90]. Prolonged infusions of GPI are common practice; however, clinical studies show that a bolus-only strategy reduces vascular/bleeding complications with a similar MACE rate compared to prolonged infusion in PCI [91,92]. However, there is no evidence in pPCI for a bolus-only strategy.

Routes of GPI infusion (intracoronary verses intravenous) have been investigated. The AIDA-STEMI trial found no disparity in primary outcomes (death, new MI, or new-onset heart failure) at 3 months between patients receiving intracoronary versus intravenous abciximab. Similarly, the Cardiac-MRI sub-study and the CICERO trial supported these findings, showing comparable results in terms of infarct size, myocardial salvage index, microvascular obstruction, and ejection fraction between the two routes [93]. Conversely, the INFUSE-AMI trial showed a significant reduction in infarct size in the intracoronary abciximab arm, possibly driven by patient selection as the study only included patients presenting early (time < 4 h) with large anterior STEMIs [94].

Currently European and American guidelines underscore that there is no strong evidence for the routine use of GPI in STEMI patients and that their use should be considered for bailout if there is evidence of no-reflow or thrombotic complications.

## 7. Intravascular Imaging of the Infarct-Related Artery

The role of intravascular imaging in pPCIs is another potential under-explored field. STEMI patients are typically under-represented in RCTs exploring the role of intracoronary imaging in PCI optimization [95,96,97].

Despite this, there is large scope and impact that intracoronary imaging can have in STEMI care. Intracoronary imaging before stent implant could play a role in defining the mechanism of plaque instability, defining the presence of significant calcified plaque requiring additional lesion preparation and assessing and quantifying the thrombotic burden. This last aspect could be helpful in defining whether further thrombus-modification techniques (device- or drug-based) should be applied and whether stenting deferral should be considered to avoid severe distal embolization and no-reflow [98].

Post-PCI intravascular imaging can reveal stent under-expansion, malposition, or edge dissections, which occur in up to one third of STEMI cases [99]. OCT studies have clearly shown a high rate of stent under expansion and stent malposition in STEMI patient follow-up. The increased vascular tone and the presence of thrombus during the index procedure can indeed contribute to stent under-sizing and malposition as thrombus resorbs and normal vascular tone is restored [100,101].

The recent IVUS-ACS trial, using intravascular imaging in acute coronary syndromes (28% STEMI), showed a lower rate of composite cardiovascular outcomes, mainly driven by target-vessel revascularisation, compared to angiography guidance alone [102].

Primary PCI is often perceived as a fast procedure and sometimes stent optimization is sacrificed in the name of speed, especially when dealing with a sick patient. As evidence is accumulating on the clinical benefit and cost-effectiveness of intracoronary imaging in routine PCI, there should be a call to investigate if there is added benefit to utilising intracoronary imaging in the context of pPCI.

## 8. DEB/DCB in STEMI

Drug-coated balloons (DCBs) and drug-eluting balloons (DEBs) have shown safety and efficacy in treating in-stent restenosis, small-vessel disease, and high-bleeding risk cohorts [103,104]. DEB-only pPCI is emerging as a feasible and safe treatment option for patients ineligible for drug-eluting stents [105]. A recent study indicated no disparity in mortality or target lesion revascularization between DCBs and DESs in pPCI patients [106]. DCBs could be beneficial in lesions with a large thrombotic burden, when employing a deferred stenting approach, or in spastic arteries where the chance of implanting an undersized and malposed stent is higher.

The implementation of DEBs/DCBs in pPCI is certainly a new line of research and numerous studies are underway, with the results eagerly awaited.

## 9. Invasive Strategy in Late-Presenting ST-Segment Elevation Myocardial Infarction: How Late Is Too Late?

The incidence of late STEMI presenters seeking medical attention more than 12 h after symptom onset ranges between 10 and 20%, according to registries [107,108]. This subgroup of patients is known to have a higher rate of hemodynamic instability, a worse chance of systolic function recovery, and higher rates of acute heart failure [109].

European and American guidelines are concordant in recommending routine immediate angiography and pPCI in STEMI patients admitted within 12 h of symptom onset. The value of a routine pPCI strategy in late-presenting STEMI patients (>12 h after symptom onset) is still not fully established.

Randomised clinical trials have established that STEMI patients presenting 12–48 h after symptom onset and without persistent symptoms of ongoing ischemia might still benefit from reperfusion therapy, as reflected by lower rates of in-hospital death and re-infarction, compared to a conservative management strategy [110]. Similarly, real-world data from multicentre registries have highlighted that late STEMI presenters who are invasively managed show a lower mortality rate both in-hospital and at five years of follow-up [108].

On the other hand, different results have emerged in very late STEMI presenters (total ischaemic time > 48 h). Hemodynamically stable patients without ongoing ischemia symptoms, despite the persistent occlusion of the culprit artery, obtain no clinical benefit from an invasive strategy in terms of mortality and hard cardiovascular endpoints [111,112,113].

There is evidence showing that, despite late presenters having a larger infarct size than early presenters, a significant amount of myocardial salvage is achievable in later presenters (beyond 12 h from symptom onset) [114]. Advanced imaging modalities such as cardiac magnetic resonance imaging (cardiac MRI) may play a key role in stratifying late presenters, defining upfront who could benefit from revascularisation and hence who should be considered for an invasive strategy, possibly irrespectively of overall ischaemic time. Indeed, there is initial evidence suggesting that STEMI patients with symptom onset within 12–72 h and signs of ongoing ischemia benefited from early revascularisation in terms of myocardial salvage, despite having larger final infarct size and a higher rate of MVO [115].

Additionally, novel CMR mapping techniques may allow a better characterization and quantification of the severity of myocardial injury as well as the extent of recoverable myocardium [116,117]. This could pave the way to a new line of research looking into a multi-parametric assessment for the risk stratification of late presenters, aiding in defining those requiring revascularisation and detecting early those with an increased chance of long-term heart failure and mechanical/arrhythmic complications.

## 10. Coronary No-Reflow: How to Diagnose It?

Despite pPCI restoring the patency of the infarct-related artery in STEMI patients, up to 40–50% experience the “no-reflow” phenomenon due to coronary microvascular dysfunction (CMD) [118]. The no-reflow phenomenon remains one of the most common complications in pPCI, significantly impacting outcomes, increasing infarct size, mortality risk, and heart failure readmission [119,120]. No-reflow arises from complex interactions including ischemic and reperfusion-related injuries, along with the embolization of athero-thrombotic material. The early diagnosis, risk stratification, and effective prevention/treatment of no-reflow pose ongoing challenges for clinicians and researchers [121,122].

Diagnosing no-reflow, the early detection of patients at higher risk of no-reflow, and preventing/treating no-reflow are still open challenges that clinicians and researchers are called to address in the near future.

The no-reflow phenomenon is usually defined on angiography as the occurrence of thrombolysis in myocardial infarction (TIMI) flow < 3 or TIMI 3 in the presence of suboptimal myocardial blush grade (MBG < 2). However, the angiographic no-reflow represents only the tip of the iceberg, as invasive angiography is suboptimal in detecting the occurrence of microvascular injury, with TIMI flow and MBG being limited by their semi-quantitative nature, poor reproducibility, and low accuracy [120,123].

The gold standard method to define the occurrence of microvascular injury in STEMI patients is cardiac magnetic resonance (cMRI), which can detect and quantify infarct size, the occurrence of microvascular obstruction, and intra-myocardial haemorrhage, which results from an advanced and irreversible degree of microvascular injury [124]. All these parameters are strongly related with clinical outcomes in STEMI patients [125]. Cardiac MRI is, however, limited by its availability/accessibility and costs. Furthermore, a cMRI-based diagnosis of microvascular injury is typically late (2–3 days after the index event) and outside the time window when additional therapy can be offered in the catheterization laboratory during or immediately after pPCI.

A new line of investigation, for this reason, has aimed at assessing the role of pressure-sensor wire technologies to assess in real time, in the catheter laboratory during pPCI, the status of coronary microcirculation. This approach would aim to detect no-reflow early (when it is not angiographically apparent) and risk-stratify STEMI patients.

Among the many indices of coronary physiology, the index of microvascular resistance (IMR) is the one most explored in the STEMI setting. IMR is the product of distal coronary pressure and the average transit time of three boluses of room-temperature saline injected in the coronary artery under assessment. Measurements are performed during steady-state hyperaemia [126].

IMR has been shown to be higher in patients with MVO [127]. IMR > 40 has been associated with left-ventricular dysfunction, higher mortality, and heart failure hospitalizations [128,129]. However, at least one third of STEMI patients exhibit a discordance between cMRI-detected MVO and IMR [130]. Whilst a co-presence of both MVO and IMR > 40 is associated with larger infarct size and strong cardiovascular events at long-term follow-up, patients with IMR < 40, even in the presence of MVO, showed better clinical outcomes and a significant regression of CMR-based infarct size at the six-month follow-up [130]. IMR measurement has been shown to be feasible before stent insertion during pPCI, with the potential to define, alone or in combination with other clinical/procedural parameters, the risk of subsequent intra-procedural post-stenting no-reflow. Thus, IMR may be a possible tool to triage high-risk patients to additional/alternative therapies in STEMI, aiming to prevent no-reflow [118,131,132].

Interestingly, besides its potential in defining high-risk STEMI patients, IMR has actually proven to have a superior ability in defining low-risk patients, with the potential to detect with high accuracy STEMI patients at a low risk of intra-hospital complications who could possibly benefit from a shorter admission [133].

One of the limitations of IMR, as of many other coronary physiology indices, is represented by its invasive nature, relying on the instrumentation of the IRA with a pressure-sensor wire. This is certainly a limiting factor towards a broader implementation of IMR in pPCI as it requires additional procedural time, costs, and procedural risks. Various versions of angiography-derived IMR have been proposed in recent years, all showing good diagnostic accuracy in detecting an abnormal IMR (comparable to its invasive counterpart), and good correlations with clinical outcomes and MVO detection using cMRI in STEMI patients [134,135]. More recently, angiography-derived IMR (namely, IMR_angio_) has shown a 98% negative predictive value in ruling out the occurrence of in-hospital and 30-day adverse events in STEMI patients, configuring itself as a promising tool to non-invasively risk-stratify STEMI patients [136].

**Figure 2:**
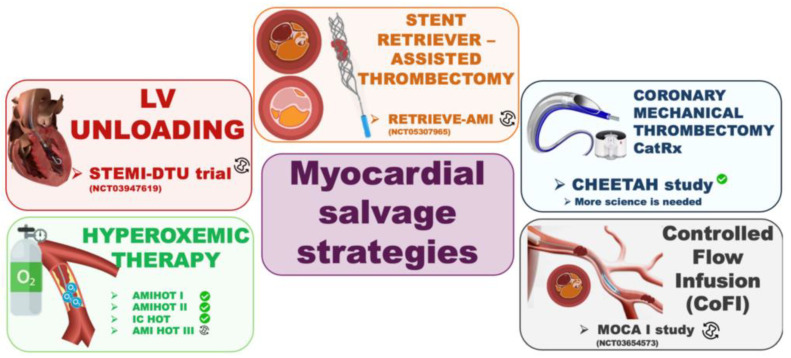
Myocardial salvage strategies. Abbreviations: LV: Left Ventricle; STEMI-DTU = Primary Unloading and Delayed Reperfusion in ST-Elevation Myocardial Infarction trial; RETRIEVE-AMI = Stent Retriever Thrombectomy for Thrombus Burden Reduction in Patients with Acute Myocardial Infarction; CHEETAH = Sustained Mechanical Aspiration Thrombectomy for High Thrombus Burden Coronary Vessel Occlusion; CoFI = Controlled Flow Infusion; MOCA I = Microvascular Obstruction With CoFI™ System Assessment; AMI-HOT = Acute Myocardial Infarction with Hyperoxemic Therapy; IC-HOT = Evaluation of Intracoronary Hyperoxemic Oxygen Therapy in Anterior Acute Myocardial Infarction Patients; NCT = National Clinical Trial number.

The implementation of these novel indices in the catheter laboratory to risk-stratify STEMI patients early could perhaps identify patients at a risk of no-reflow and guide treatment strategy in the upcoming years (Figure 2).

## 11. Coronary No-Reflow: How to Treat and Prevent It?

The prevention and treatment of coronary no-reflow after pPCI represents a major outstanding challenge in STEMI care. Several strategies (device- and drug-based) have been tested over the past 20 years, but consistently with negative or neutral results. When it comes to additional therapy for no-reflow prevention/therapy, a typical pattern is consistently observed. Strategies with promising initial results on surrogate outcomes in pilot/proof-of-concept studies then fail to deliver when stress-tested in properly powered randomised clinical trials. It is likely that suboptimal patient selection, with an “all-comers” approach testing the routine implementation of additional therapies in STEMI patients, might have played a role in undermining the true ability of trials to prove the additional clinical benefit of new therapies during or after pPCI.

When it comes to device-based therapies, the only one demonstrating any efficacy so far is represented by the intracoronary delivery of supersaturated oxygen (SSO_2_) (e.g., hyperoxemic blood with P_a_O2 760–1000 mmHg). This approach has been shown in animal models to reduce oedematous capillary swelling and to improves microcirculatory function [137]. The safety and feasibility of SSO2 were demonstrated in the AMIHOT I study, whilst initial efficacy on infarct size reduction (assessed tc-99m sestamibi SPECT) was demonstrated in the AMIHOT II study. The AMIHOT III study (NCT04743245) is a post-market trial now underway, randomising 434 patients with anterior STEMI to 60 min of intracoronary SSO2 post pPCI versus conventional pPCI, and powered to show the superiority of the treatment arm for the 1-year rate of net adverse clinical events.

The mechanical unloading of the left ventricle before coronary reperfusion is another possibly ground-breaking concept. Animal models have shown that mechanical unloading before reperfusion could reduce left ventricular work and infarct size at 30 days by activating molecular pathways protecting against reperfusion injury [138,139]. In this regard, unloading with mechanical circulatory support (Impella CP) before reperfusion has proven to be safe and feasible in humans. In the DTU-PILOT study, in anterior STEMI patients, reperfusion was delayed to allow unloading first [140]. Whether unloading the ventricle before reperfusion can now translate into an actual benefit and reduction in infarct size in humans, is the object of the ongoing STEMI-DTU trial (NCT03947619), aiming at randomising 668 patients with anterior STEMI to 30 min of Impella before reperfusion versus conventional pPCI [141].

The prevention of distal embolization by dealing upstream with large thrombus burden has appeared from the start as an obvious strategy associated with potential clinical benefit. However, after the initial positive results associated with manual thrombus aspiration [142], subsequent larger and ad hoc powered randomized clinical trials have failed to demonstrate a clinical benefit associated with routine manual thrombus aspiration during pPCI [143,144].

Besides limitations related with patient selection bias, the reason why manual thrombus aspiration might have failed to show some benefit could also be related with its actual ability in reducing thrombotic burden. The OCT sub-study of the TOTAL trial surprisingly showed no difference in post-PCI thrombus burden between the thrombus aspiration group and control group [145]. At the same time, the rate of aspiration failure remains high after manual thrombus aspiration, and a higher residual thrombus burden relates with more severe microvascular dysfunction [146]. A renewed interest in coronary thrombectomy has been reignited by advances made in stroke interventions with clot retrievers. A few case-reports have suggested that these new devices could be superior at entangling large coronary thrombi, offering a circumferential interaction with the thrombus. The RETRIEVE-AMI study (NCT05307965) is currently ongoing, aimed at assessing the difference in thrombus-burden reduction with stent-retriever thrombectomy compared to manual thrombus aspiration in STEMI [147].

A new generation of aspiration catheters (CatRX, Penumbra) has been developed which may offer greater thrombus extraction. The CatRX system provides steady and sustained aspiration, in contrast to manual aspiration which allows only short-lived suction that diminishes overtime. The CHEETAH registry has confirmed the feasibility of this technique, with promising results in terms of the low rate of angiographic no-reflow [148]. Whether this technology can offer superior thrombus extraction to manual aspiration and/or an actual benefit in terms of infarct size or MVO reduction remains to be seen.

Evidence for drug therapy to prevent or treat no-reflow is minimal. To date, evidence is conflicting for several classes of medicine aiming to manage no-reflow [149]. To cite two examples: intracoronary thrombolytics were shown to be ineffective in the T-TIME trial [150], whilst intracoronary adenosine in the REOPEN-AMI study did show benefit in that MVO was reduced compared to placebo [151].

It has been hypothesized that besides the agent adopted, the way it is delivered may also play a role. A new technology, the CoFI system (Corflow) consists of a dedicated infusion catheter, with a balloon at its tip to occlude coronary flow during infusion. This approach results in higher tissue drug retention compared to coronary infusion via a microcatheter or via a guiding catheter. The system also has diagnostic capabilities. By measuring distal coronary pressure at increasing infusion rates of saline, it can provide an assessment of coronary microvascular function, with the potential to risk-stratify STEMI patients, integrating the early detection of no-reflow with ad hoc therapy. This technology, however, remains at the early stages of development. The MOCA I study (NCT03654573) is collecting initial safety and feasibility data and preliminary data on the impact on infract size and MVO with intracoronary tirofiban using the CoFI system.

## 12. Conclusions

STEMI care has certainly evolved over the past three decades, with the undoubted impact of pPCI on survival and quality of life for STEMI patients. However, STEMI care is far from optimal. This is reflected by the plateau in clinical outcomes in STEMI and the increasing burden of heart failure in STEMI survivors.

Research in this field remains active and endeavours are underway to reduce symptom-to-balloon time, define novel/alternative care pathways, and explore additional diagnostic and therapeutic tools to prevent or deal with coronary no-reflow. This review has highlighted the main upcoming lines of investigation in the field, and there is an expectation and hope that results from ongoing research might reshape the landscape of STEMI care in the upcoming years.

## References

[1] Hartzler G.O., Rutherford B.D., McConahay D.R., Johnson W.L., McCallister B.D., Gura G.M., Conn R.C., Crockett J.E. (1983). Percutaneous transluminal coronary angioplasty with and without thrombolytic therapy for treatment of acute myocardial infarction. Am. Heart J..

[2] Hartzler G.O., Rutherford B.D., McConahay D.R. (1984). Percutaneous Transluminal Coronary Angioplasty: Application for Acute Myocardial Infarction. Am. J. Cardiol..

[3] Topol E.J., Califf R.M., George B.S., Kereiakes D.J., Abbottsmith C.W., Candela R.J., Lee K.L., Pitt B., Stack R.S., O’Neill W.W. (1987). A randomized trial of immediate versus delayed elective angioplasty after intravenous tissue plasminogen activator in acute myocardial infarction. N. Engl. J. Med..

[4] Simoons M.L., Betriu A., Col J., Von Essen R., Lubsen J., Michel P.L., Rutsch W., Schmidt W., Thery C., Vahanian A. (1988). Saturday 30 January 1988 thrombolysis with tissue plasminogen activator in acute myocardial infarction: No additional benefit from immediate percutaneous coronary angioplasty. Lancet.

[5] O’Keefe J.H., Rutherford B.D., McConahay D.R., Ligon R.W., Johnson W.L., Giorgi L.V., Crockett J.E., McCallister B.D., Conn R.D., Gura G.M. (1989). Early and Late Results of Coronary Angioplasty without Antecedent Thrombolytic Therapy for Acute Myocardial Infarction. Am. J. Cardiol..

[6] O’Neill W.W., Brodie B.R., Ivanhoe R., Knopf W., Taylor G., O’Keefe J., Grines C.L., Weintraub R., Sickinger B.G., Berdan L.G. (1994). Cardiology Primary Coronary Angioplasty for Acute Myocardial Infarction (the Primary Angioplasty Redstry). Am. J. Cardiol..

[7] Holmes D.R., Vlietstra R.E., Smith H.C., Vetrovec G.W., Kent K.M., Cowley M.J., Faxon D.P., Gruentzig A.R., Kelsey S.F., Detre K.M. (1984). Restenosis After Percutaneous Transluminal Coronary Angioplasty (PTCA): A Report from the PTCA Registry of the National Heart, Lung, and Blood Institute. Am. J. Cardiol..

[8] Meier B., King S.B., Gruentzig A.R., Douglas J.S., Hollman J., Ischinger T., Galan K., Tankersley R. (1984). Repeat coronary angioplasty. J. Am. Coll. Cardiol..

[9] Schatz R.A., Baim D.S., Leon M., Ellis S.G., Goldberg S., Hirshfeld J.W., Cleman M.W., Cabin H.S., Walker C., Stagg J. (1991). Clinical Experience with the Palmaz-Schatz Coronary Stent Initial Results of a Multicenter Study. Circulation.

[10] Fischman D.L., Leon M.B., Baim D.S., Schatz R.A., Savage M.P., Penn I., Detre K., Veltri L., Ricci D., Nobuyoshi M. (1994). A randomized comparison of coronary-stent placement and balloon angioplasty in the treatment of coronary artery disease. Stent Restenosis Study Investigators. N. Engl. J. Med..

[11] Serruys P.W., van Hout B., Bonnier H., Legrand V., Garcia E., Macaya C., Sousa E., van der Giessen W., Colombo A., Seabra-Gomes R. (1998). Randomised comparison of implantation of heparin-coated stents with balloon angioplasty in selected patients with coronary artery disease (Benestent II). Lancet.

[12] Antoniucci D., Santoro G.M., Bolognese L., Valenti R., Trapani M., Fazzini P.F. (1998). A Clinical Trial Comparing Primary Stenting of the Infarct-Related Artery with Optimal Primary Angioplasty for Acute Myocardial Infarction Results From the Florence Randomized Elective Stenting in Acute Coronary Occlusions (FRESCO) Trial. J. Am. Coll. Cardiol..

[13] Stone G.W., Grines C.L., Cox D.A., Garcia E., Tcheng J.E., Griffin J.J., Guagliumi G., Stuckey T., Turco M., Carroll J.D. (2002). Controlled Abciximab and Device Investigation to Lower Late Angioplasty Complications (CADILLAC) Investigators. Comparison of angioplasty with stenting, with or without abciximab, in acute myocardial infarction. N. Engl. J. Med..

[14] Grines C.L., Cox D.A., Stone G.W., Garcia E., Mattos L.A., Giambartolomei A., Brodie B.R., Madonna O., Eijgelshoven M., Lansky A.J. (1999). Coronary angioplasty with or without stent implantation for acute myocardial infarction. Stent Primary Angioplasty in Myocardial Infarction Study Group. N. Engl. J. Med..

[15] Stone G.W., Ellis S.G., Cox D.A., Hermiller J., O’Shaughnessy C., Mann J.T., Turco M., Caputo R., Bergin P., Greenberg J. (2004). A polymer-based, paclitaxel-eluting stent in patients with coronary artery disease. N. Engl. J. Med..

[16] Mauri L., Silbaugh T.S., Garg P., Wolf R.E., Zelevinsky K., Lovett A., Varma M.R., Zhou Z., Normand S.L. (2008). Drug-eluting or bare-metal stents for acute myocardial infarction. N. Engl. J. Med..

[17] Daemen J., Tanimoto S., García-García H.M., Kukreja N., van de Sande M., Sianos G., de Jaegere P.P., van Domburg R.T., Serruys P.W. (2007). Comparison of Three-Year Clinical Outcome of Sirolimus- and Paclitaxel-Eluting Stents Versus Bare Metal Stents in Patients with ST-Segment Elevation Myocardial Infarction (from the RESEARCH and T-SEARCH Registries). Am. J. Cardiol..

[18] Piccolo R., Stefanini G.G., Franzone A., Spitzer E., Blöchlinger S., Heg D., Jüni P., Windecker S. (2015). Safety and Efficacy of Resolute Zotarolimus-Eluting Stents Compared with Everolimus-Eluting Stents. Circ. Cardiovasc. Interv..

[19] Jensen L.O., Thayssen P., Christiansen E.H., Maeng M., Ravkilde J., Hansen K.N., Hansen H.S., Krusell L., Kaltoft A., Tilsted H.H. (2016). Safety and Efficacy of Everolimus-Versus Sirolimus-Eluting Stents 5-Year Results From SORT OUT IV. J. Am. Coll. Cardiol..

[20] Iglesias J.F., Roffi M., Losdat S., Muller O., Degrauwe S., Kurz D.J., Haegeli L., Weilenmann D., Kaiser C., Tapponnier M. (2023). Long-term outcomes with biodegradable polymer sirolimus-eluting stents versus durable polymer everolimus-eluting stents in ST-segment elevation myocardial infarction: 5-year follow-up of the BIOSTEMI randomised superiority trial. Lancet.

[21] Serruys P.W., Chevalier B., Sotomi Y., Cequier A., Carrié D., Piek J.J., Van Boven A.J., Dominici M., Dudek D., McClean D. (2016). Comparison of an everolimus-eluting bioresorbable scaff old with an everolimus-eluting metallic stent for the treatment of coronary artery stenosis (ABSORB II): A 3 year, randomised, controlled, single-blind, multicentre clinical trial. Lancet.

[22] Kereiakes D.J., Ellis S.G., Metzger D.C., Caputo R.P., Rizik D.G., Teirstein P.S., Litt M.R., Kini A., Kabour A., Marx S.O. (2019). Clinical Outcomes before and after Complete Everolimus-Eluting Bioresorbable Scaffold Resorption: Five-Year Follow-Up from the ABSORB III Trial. Circulation.

[23] Karrowni W., Vyas A., Giacomino B., Schweizer M., Blevins A., Girotra S., Horwitz P.A. (2013). Radial Versus Femoral Access for Primary Percutaneous Interventions in ST-Segment Elevation Myocardial Infarction Patients A Meta-Analysis of Randomized Controlled Trials. JACC Cardiovasc. Interv..

[24] Mori H., Sakurai K., Ikari Y., Fukui K., Maeda A., Akashi Y., Ako J., Ebina T., Tamura K., Namiki A. (2023). Radial versus femoral access in patients undergoing primary percutaneous coronary intervention for ST-elevation myocardial infarction: A propensity-matched analysis from real-world data of the K-ACTIVE registry. J. Cardiol..

[25] Steg P.G., Goldberg R.J., Gore J.M., Fox K.A., Eagle K.A., Flather M.D., Sadiq I., Kasper R., Rushton-Mellor S.K., Anderson F.A. (2002). Baseline characteristics, management practices, and in-hospital outcomes of patients hospitalized with acute coronary syndromes in the Global Registry of Acute Coronary Events (GRACE). Am. J. Cardiol..

[26] Salari N., Morddarvanjoghi F., Abdolmaleki A., Rasoulpoor S., Khaleghi A.A., Hezarkhani L.A., Shohaimi S., Mohammadi M. (2023). The global prevalence of myocardial infarction: A systematic review and meta-analysis. BMC Cardiovasc. Disord..

[27] Schmidt M., Jacobsen J.B., Lash T.L., Bøtker H.E., Sørensen H.T. (2012). 25 Year trends in first time hospitalisation for acute myocardial infarction, subsequent short and long term mortality, and the prognostic impact of sex and comorbidity: A Danish nationwide cohort study. BMJ.

[28] Krishnan U., Brejt J.A., Schulman-Marcus J., Swaminathan R.V., Feldman D.N., Goyal P., Wong S.C., Minutello R.M., Bergman G., Singh H. (2018). Temporal Trends in the Clinical Acuity of Patients with ST-Segment Elevation Myocardial Infarction. Am. J. Med..

[29] Puymirat E., Simon T., Cayla G., Cottin Y., Elbaz M., Coste P., Lemesle G., Motreff P., Popovic B., Khalife K. (2017). Acute myocardial infarction: Changes in patient characteristics, management, and 6-month outcomes over a period of 20 years in the FAST-MI program (French registry of acute ST-elevation or non-ST-elevation myocardial infarction) 1995 to 2015. Circulation.

[30] Fokkema M.L., James S.K., Albertsson P., Akerblom A., Calais F., Eriksson P., Jensen J., Nilsson T., de Smet B.J., Sjögren I. (2013). Population trends in percutaneous coronary intervention: 20-Year Results: From the SCAAR (Swedish Coronary Angiography and Angioplasty Registry). J. Am. Coll. Cardiol..

[31] Pedersen F., Butrymovich V., Kelbæk H., Wachtell K., Helqvist S., Kastrup J., Holmvang L., Clemmensen P., Engstrøm T., Grande P. (2014). Short-and Long-Term Cause of Death in Patients Treated with Primary PCI for STEMI. J. Am. Coll. Cardiol..

[32] Yan F., Zhang Y., Pan Y., Li S., Yang M., Wang Y., Yanru C., Su W., Ma Y., Han L. (2023). Prevalence and associated factors of mortality after percutaneous coronary intervention for adult patients with ST-elevation myocardial infarction: A systematic review and meta-analysis. J. Res. Med Sci..

[33] Grines C.L., Browne K.F., Marco J., Rothbaum D., Stone G.W., O’Keefe J., Overlie P., Donohue B., Chelliah N., Timmis G.C. (1993). A comparison of immediate angioplasty with thrombolytic therapy for acute myocardial infarction. The Primary Angioplasty in Myocardial Infarction Study Group. N. Engl. J. Med..

[34] Costa R., Trêpa M., Oliveira M., Frias A., Campinas A., Luz A., Santos M., Torres S. (2022). Heart Failure Incidence Following ST-Elevation Myocardial Infarction. Am. J. Cardiol..

[35] Reimer K.A., Lowe J.E., Rasmussen M.M., Jennings R.B. (1977). The Wavefront Phenomenon of Ischemic Cell Death 1. Myocardial Infarct Size vs Duration of Coronary Occlusion in Dogs. Circulation.

[36] Hasche E.T., Fernandes C., Freedman S.B., Jeremy R.W. (1995). Relation between ischemia time, infarct size, and left ventricular function in humans. Circulation.

[37] Tarantini G., Cacciavillani L., Corbetti F., Ramondo A., Marra M.P., Bacchiega E., Napodano M., Bilato C., Razzolini R., Iliceto S. (2005). Duration of ischemia is a major determinant of transmurality and severe microvascular obstruction after primary angioplasty: A study performed with contrast-enhanced magnetic resonance. J. Am. Coll. Cardiol..

[38] Menees D.S., Peterson E.D., Wang Y., Curtis J.P., Messenger J.C., Rumsfeld J.S., Gurm H.S. (2013). Door-to-Balloon Time and Mortality among Patients Undergoing Primary PCI. N. Engl. J. Med..

[39] Nallamothu B.K., Normand S.-L.T., Wang Y., Hofer T.P., Brush J.E., Messenger J.C., Bradley E.H., Rumsfeld J.S., Krumholz H.M. (2014). Relation between door-to-balloon times and mortality after primary percutaneous coronary intervention over time: A retrospective study. Lancet.

[40] Denktas A.E., Anderson H.V., McCarthy J., Smalling R.W. (2011). MINI-FOCUS ISSUE: STEMI State-of-the-Art Paper Total Ischemic Time The Correct Focus of Attention for Optimal ST-Segment Elevation Myocardial Infarction Care. JACC Cardiovasc. Interv..

[41] Doll J.A., Roe M.T. (2015). Time to treatment as a quality metric for acute STEMI care. Lancet.

[42] Zijlstra F., Patel A.A., Jones M., Grines C., Ellis S., Garcia E.E., Grinfeld L., Gibbons R., Ribeiro E., Ribichini F. (2002). Clinical characteristics and outcome of patients with early (<2 h), intermediate (2–4 h) and late (>4 h) presentation treated by primary coronary angioplasty or thrombolytic therapy for acute myocardial infarction. Eur. Heart J..

[43] De Luca G., Suryapranata H., Ottervanger J.P., Antman E.M. (2004). Time Delay to Treatment and Mortality in Primary Angioplasty for Acute Myocardial Infarction: Every Minute of Delay Counts. Circulation.

[44] Chandrasekhar J., Marley P., Allada C., McGill D., O’Connor S., Rahman M., Tan R., Hosseiny A.D., Shadbolt B., Farshid A. (2017). Symptom-to-Balloon Time is a Strong Predictor of Adverse Events Following Primary Percutaneous Coronary Intervention: Results From the Australian Capital Territory PCI Registry. Heart Lung Circ..

[45] De Luca G., Silverio A., Verdoia M., Siudak Z., Tokarek T., Kite T.A., Gershlick A.H., Rodriguez-Leor O., Cid-Alvarez B., Jones D.A. (2022). Angiographic and clinical outcome of SARS-CoV-2 positive patients with ST-segment elevation myocardial infarction undergoing primary angioplasty: A collaborative, individual patient data meta-analysis of six registry-based studies. Eur. J. Intern. Med..

[46] Farshid A., Allada C., Chandrasekhar J., Marley P., McGill D., O’Connor S., Rahman M., Tan R., Shadbolt B. (2015). Shorter ischaemic time and improved survival with pre-hospital STEMI diagnosis and direct transfer for primary PCI. Heart Lung Circ..

[47] Thylén I., Ericsson M., Ängerud K.H., Isaksson R.M., Lawesson S.S. (2015). First medical contact in patients with STEMI and its impact on time to diagnosis; an explorative cross-sectional study. BMJ Open.

[48] Stopyra J.P., Snavely A.C., Ashburn N.P., Supples M.W., Miller C.D., Mahler S.A. (2023). Delayed first medical contact to reperfusion time increases mortality in rural emergency medical services patients with ST-elevation myocardial infarction. Acad. Emerg. Med..

[49] Bishu K.G., Lekoubou A., Kirkland E., Schumann S.O., Schreiner A., Heincelman M., Moran W.P., Mauldin P.D. (2020). Estimating the Economic Burden of Acute Myocardial Infarction in the US: 12 Year National Data. Am. J. Med Sci..

[50] Rathod K.S., Koganti S., Iqbal M.B., Jain A.K., Kalra S.S., Astroulakis Z., Lim P., Rakhit R., Dalby M., Lockie T. (2018). Contemporary trends in cardiogenic shock: Incidence, intra-aortic balloon pump utilisation and outcomes from the London Heart Attack Group. Eur. Heart J. Acute Cardiovasc. Care.

[51] Thiele H., Akin I., Sandri M., Fuernau G., De Waha S., Meyer-Saraei R., Nordbeck P., Geisler T., Landmesser U., Skurk C. (2017). PCI Strategies in Patients with Acute Myocardial Infarction and Cardiogenic Shock. N. Engl. J. Med..

[52] Thiele H., Zeymer U., Neumann F.-J., Ferenc M., Olbrich H.-G., Hausleiter J., Richardt G., Hennersdorf M., Empen K., Fuernau G. (2012). Intraaortic Balloon Support for Myocardial Infarction with Cardiogenic Shock. N. Engl. J. Med..

[53] Hochman J.S., Sleeper L.A., White H.D., Dzavik V., Wong S.C., Menon V., Webb J.G., Steingart R., Picard M.H., Menegus M.A. (2001). Should We Emergently Revascularize Occluded Coronaries for Cardiogenic Shock. One-year survival following early revascularization for cardiogenic shock. JAMA.

[54] Thiele H., Zeymer U., Neumann F.-J., Ferenc M., Olbrich H.-G., Hausleiter J., de Waha A., Richardt G., Hennersdorf M., Empen K. (2013). Intra-aortic balloon counterpulsation in acute myocardial infarction complicated by cardiogenic shock (IABP-SHOCK II): Final 12 month results of a randomised, open-label trial. Lancet.

[55] Hochman J.S., Sleeper L.A., Webb J.G., Sanborn T.A., White H.D., Talley J.D., Buller C.E., Jacobs A.K., Slater J.N., Col J. (1999). Should We Emergently Revascularize Occluded Coronaries for Cardiogenic Shock. N. Engl. J. Med..

[56] Hochman J.S., Sleeper L.A., Webb J.G., Dzavik V., Buller C.E., Aylward P., Col J., White H.D. (2006). Early Revascularization and Long-term Survival in Cardiogenic Shock Complicating Acute Myocardial Infarction. JAMA.

[57] Jeger R.V., Radovanovic D., Hunziker P.R., Pfisterer M.E., Stauffer J.C., Erne P., Urban P., AMIS Plus Registry Investigators (2008). Ten-year trends in the incidence and treatment of cardiogenic shock. Ann. Intern. Med..

[58] Kochar A., Al-Khalidi H.R., Hansen S.M., Shavadia J.S., Roettig M.L., Fordyce C.B., Doerfler S., Gersh B.J., Henry T.D., Berger P.B. (2018). Delays in Primary Percutaneous Coronary Intervention in ST-Segment Elevation Myocardial Infarction Patients Presenting with Cardiogenic Shock. JACC Cardiovasc. Interv..

[59] Sanborn T.A., Sleeper L.A., Webb J.G., French J.K., Bergman G., Parikh M., Wong S., Boland J., Pfisterer M., Slater J.N. (2003). Correlates of One-Year Survival in Patients with Cardiogenic Shock Complicating Acute Myocardial Infarction: Angiographic Findings from the SHOCK Trial. J. Am. Coll. Cardiol..

[60] Gerbaud E., Elbaz M., Lattuca B. (2020). New insights into cardiogenic shock and coronary revascularization after acute myocardial infarction. Arch. Cardiovasc. Dis..

[61] Thiele H., Akin I., Sandri M., De Waha-Thiele S., Meyer-Saraei R., Fuernau G., Eitel I., Nordbeck P., Geisler T., Landmesser U. (2018). One-Year Outcomes after PCI Strategies in Cardiogenic Shock. N. Engl. J. Med..

[62] Kochar A., Varshney A.S., Wang D.E. (2021). Residual SYNTAX Score After Revascularization in Cardiogenic Shock: When Is Complete Complete?. J. Am. Coll. Cardiol..

[63] Lee J.M., Rhee T., Kim H.K., Hwang D., Lee S.H., Choi K.H., Kim J., Park T.K., Yang J.H., Bin Song Y. (2019). Comparison of Long-Term Clinical Outcome Between Multivessel Percutaneous Coronary Intervention Versus Infarct-Related Artery-Only Revascularization for Patients with ST-Segment-Elevation Myocardial Infarction with Cardiogenic Shock. J. Am. Heart Assoc..

[64] Lee J.M., Rhee T.-M., Hahn J.-Y., Kim H.K., Park J., Hwang D., Choi K.H., Kim J., Park T.K., Yang J.H. (2018). Multivessel Percutaneous Coronary Intervention in Patients with ST-Segment Elevation Myocardial Infarction with Cardiogenic Shock. J. Am. Coll. Cardiol..

[65] Thiele H., Zeymer U., Thelemann N., Neumann F.-J., Hausleiter J., Abdel-Wahab M., Meyer-Saraei R., Fuernau G., Eitel I., Hambrecht R. (2019). Intraaortic Balloon Pump in Cardiogenic Shock Complicating Acute Myocardial Infarction: Long-Term 6-Year Outcome of the Randomized IABP-SHOCK II Trial. Circulation.

[66] Basir M.B., Schreiber T.L., Grines C.L., Dixon S.R., Moses J.W., Maini B.S., Khandelwal A.K., Ohman E.M., O’Neill W.W. (2017). Effect of Early Initiation of Mechanical Circulatory Support on Survival in Cardiogenic Shock. Am. J. Cardiol..

[67] Ouweneel D.M., Eriksen E., Sjauw K.D., van Dongen I.M., Hirsch A., Packer E.J., Vis M.M., Wykrzykowska J.J., Koch K.T., Baan J. (2017). Percutaneous Mechanical Circulatory Support Versus Intra-Aortic Balloon Pump in Cardiogenic Shock After Acute Myocardial Infarction. J. Am. Coll. Cardiol..

[68] Møller J.E., Engstrøm T., Jensen L.O., Eiskjær H., Mangner N., Polzin A., Schulze P.C., Skurk C., Nordbeck P., Clemmensen P. (2024). Microaxial Flow Pump or Standard Care in Infarct-Related Cardiogenic Shock. N. Engl. J. Med..

[69] Schäfer A., Westenfeld R., Sieweke J.-T., Zietzer A., Wiora J., Masiero G., Martinez C.S., Tarantini G., Werner N. (2021). Complete Revascularisation in Impella-Supported Infarct-Related Cardiogenic Shock Patients Is Associated with Improved Mortality. Front. Cardiovasc. Med..

[70] Giralt T., Carrillo X., Rodriguez-Leor O., Fernandez-Nofrerias E., Rueda F., Serra-Flores J., Viguer J.M., Mauri J., Curos A., Bayes-Genis A. (2015). Time-dependent effects of unfractionated heparin in patients with ST-elevation myocardial infarction transferred for primary angioplasty. Int. J. Cardiol..

[71] Karlsson S., Andell P., A Mohammad M., Koul S., Olivecrona G.K., James S.K., Fröbert O., Erlinge D. (2019). Editor’s Choice- Heparin pre-treatment in patients with ST-segment elevation myocardial infarction and the risk of intracoronary thrombus and total vessel occlusion. Insights from the TASTE trial. Eur. Heart J. Acute Cardiovasc. Care.

[72] Emilsson O.L., Bergman S., Mohammad M.M., Olivecrona G.O., Götberg M., Erlinge D., Koul S. (2022). Pretreatment with heparin in patients with ST-segment elevation myocardial infarction: A report from the Swedish Coronary Angiography and Angioplasty Registry (SCAAR). EuroIntervention.

[73] Montalescot G., Zeymer U., Silvain J., Boulanger B., Cohen M., Goldstein P., Ecollan P., Combes X., Huber K., Pollack C. (2011). Intravenous enoxaparin or unfractionated heparin in primary percutaneous coronary intervention for ST-elevation myocardial infarction: The international randomised open-label ATOLL trial. Lancet.

[74] Li Y., Liang Z., Qin L., Wang M., Wang X., Zhang H., Liu Y., Li Y., Jia Z., Liu L. (2022). Bivalirudin plus a high-dose infusion versus heparin monotherapy in patients with ST-segment elevation myocardial infarction undergoing primary percutaneous coronary intervention: A randomised trial. Lancet.

[75] Wiviott S.D., Braunwald E., McCabe C.H., Montalescot G., Ruzyllo W., Gottlieb S., Neumann F.-J., Ardissino D., De Servi S., Murphy S.A. (2007). Prasugrel versus Clopidogrel in Patients with Acute Coronary Syndromes. N. Engl. J. Med..

[76] Wallentin L., Becker R.C., Budaj A., Cannon C.P., Emanuelsson H., Held C., Horrow J., Husted S., James S., Katus H. (2009). Ticagrelor versus Clopidogrel in Patients with Acute Coronary Syndromes. N. Engl. J. Med..

[77] Gimbel M., Qaderdan K., Willemsen L., Hermanides R., Bergmeijer T., de Vrey E., Heestermans T., Gin M.T., Waalewijn R., Hofma S. (2020). Clopidogrel versus ticagrelor or prasugrel in patients aged 70 years or older with non-ST-elevation acute coronary syndrome (POPular AGE): The randomised, open-label, non-inferiority trial. Lancet.

[78] Urban P., Mehran R., Colleran R., Angiolillo D.J., Byrne R.A., Capodanno D., Cuisset T., Cutlip D., Eerdmans P., Eikelboom J. (2019). Defining High Bleeding Risk in Patients Undergoing Percutaneous Coronary Intervention. Circulation.

[79] Montalescot G., van ‘t Hof A.W., Lapostolle F., Silvain J., Lassen J.F., Bolognese L., Cantor W.J., Cequier Á., Chettibi M., Goodman S.G. (2014). Prehospital Ticagrelor in ST-Segment Elevation Myocardial Infarction. N. Engl. J. Med..

[80] Koul S., Smith J.G., Götberg M., Omerovic E., Alfredsson J., Venetsanos D., Persson J., Jensen J., Lagerqvist B., Redfors B. (2018). No Benefit of Ticagrelor Pretreatment Compared with Treatment During Percutaneous Coronary Intervention in Patients with ST-Segment-Elevation Myocardial Infarction Undergoing Primary Percutaneous Coronary Intervention. Circ. Cardiovasc. Interv..

[81] Montalescot G., Bolognese L., Dudek D., Goldstein P., Hamm C., Tanguay J.-F., Berg J.M.T., Miller D.L., Costigan T.M., Goedicke J. (2013). Pretreatment with Prasugrel in Non–ST-Segment Elevation Acute Coronary Syndromes. N. Engl. J. Med..

[82] Vlachojannis G.J., Wilschut J.M., Vogel R.F., Lemmert M.E., Delewi R., Diletti R., van der Waarden N.W.P.L., Nuis R.-J., Paradies V., Alexopoulos D. (2020). Effect of Prehospital Crushed Prasugrel Tablets in Patients with ST-Segment-Elevation Myocardial Infarction Planned for Primary Percutaneous Coronary Intervention: The Randomized COMPARE CRUSH Trial. Circulation.

[83] Vogel R.F., Delewi R., Wilschut J.M., Lemmert M.E., Diletti R., van Vliet R., van der Waarden N.W., Nuis R.-J., Paradies V., Alexopoulos D. (2022). Pre-hospital treatment with crushed versus integral tablets of prasugrel in patients presenting with ST-Segment Elevation Myocardial Infarction—1-year follow-up results of the COMPARE CRUSH trial. Am. Heart J..

[84] Wilschut J.M., Vogel R.F., Elscot J.J., Delewi R., Lemmert M.E., van der Waarden N.W., Nuis R.-J., Paradies V., Alexopoulos D., Zijlstra F. (2024). Prehospital crushed versus integral prasugrel loading dose in STEMI patients with a large myocardial area. EuroIntervention.

[85] Bhatt D.L., Topol E.J. (2000). Current role of platelet glycoprotein IIb/IIIa inhibitors in acute coronary syndromes. JAMA.

[86] Kastrati A., Mehilli J., Neumann F.J., Dotzer F., ten Berg J., Bollwein H., Graf I., Ibrahim M., Pache J., Seyfarth M. (2006). Abciximab in patients with acute coronary syndromes undergoing percutaneous coronary intervention after clopidogrel pretreatment: The ISAR-REACT 2 randomized trial. JAMA.

[87] Boersma E., Harrington R.A., Moliterno D.J., White H., Théroux P., Van de Werf F., de Torbal A., Armstrong P.W., Wallentin L.C., Wilcox R.G. (2002). Platelet glycoprotein IIb/IIIa inhibitors in acute coronary syndromes: A meta-analysis of all major randomised clinical trials. Lancet.

[88] Topol E.J., Mark D.B., Lincoff A.M., Cohen E., Burton J., Kleiman N., Talley D., Sapp S., Booth J., Cabot C.F. (1999). Outcomes at 1 year and economic implications of platelet glycoprotein IIb/IIIa blockade in patients undergoing coronary stenting: Results from a multicentre randomised trial. Lancet.

[89] Marso S.P., Lincoff A.M., Ellis S.G., Bhatt D.L., Tanguay J.F., Kleiman N.S., Hammoud T., Booth J.E., Sapp S.K., Topol E.J. (1999). Optimizing the percutaneous interventional outcomes for patients with diabetes mellitus: Results of the EPISTENT (Evaluation of platelet IIb/IIIa inhibitor for stenting trial) diabetic substudy. Circulation.

[90] Heestermans A.A.C.M., Van Werkum J.W., Hamm C., Dill T., Gosselink A.T.M., De Boer M.J., Van Houwelingen G., Hoorntje J.C.A., Koopmans P.C., Berg J.M.T. (2009). Marked reduction of early stent thrombosis with pre-hospital initiation of high-dose tirofiban in ST-segment elevation myocardial infarction. J. Thromb. Haemost..

[91] Mehilli J., Kastrati A., Schulz S., Früngel S., Nekolla S.G., Moshage W., Dotzer F., Huber K., Pache J., Dirschinger J. (2009). Abciximab in patients with acute sT-segment-elevation myocardial infarction undergoing primary percutaneous coronary intervention after clopidogrel loading a randomized double-blind trial. Circulation.

[92] Kini A.S., Chen V.H., Krishnan P., Lee P., Kim M.C., Mares A., Suleman J., Moreno P.R., Sharma S.K. (2008). Bolus-only versus bolus + infusion of glycoprotein IIb/IIIa inhibitors during percutaneous coronary intervention. Am. Heart J..

[93] Bertrand O.F., De Larochellière R., Rodés-Cabau J., Proulx G., Gleeton O., Nguyen C.M., Déry J.-P., Barbeau G., Noël B., Larose E. (2006). A randomized study comparing same-day home discharge and abciximab bolus only to overnight hospitalization and abciximab bolus and infusion after transradial coronary stent implantation. Circulation.

[94] Eitel I., Wöhrle J., Suenkel H., Meissner J., Kerber S., Lauer B., Pauschinger M., Birkemeyer R., Axthelm C., Zimmermann R. (2013). Intracoronary compared with intravenous bolus abciximab application during primary percutaneous coronary intervention in ST-segment elevation myocardial infarction: Cardiac magnetic resonance substudy of the AIDA STEMI trial. J. Am. Coll. Cardiol..

[95] Stone G.W., Maehara A., Witzenbichler B., Godlewski J., Parise H., Dambrink J.-H.E., Ochala A., Carlton T.W., Cristea E., Wolff S.D. (2012). Intracoronary abciximab and aspiration thrombectomy in patients with large anterior myocardial infarction: The INFUSE-AMI randomized trial. JAMA.

[96] Zhang J., Gao X., Kan J., Ge Z., Han L., Lu S., Tian N., Lin S., Lu Q., Wu X. (2018). Intravascular Ultrasound Versus Angiography-Guided Drug-Eluting Stent Implantation: The ULTIMATE Trial. J. Am. Coll. Cardiol..

[97] Hong S.J., Kim B.-K., Shin D.-H., Nam C.-M., Kim J.-S., Ko Y.-G., Choi D., Kang T.-S., Kang W.-C., Her A.-Y. (2015). Effect of intravascular ultrasound-guided vs angiography- guided everolimus-eluting stent implantation: The IVUS-XPL randomized clinical trial. JAMA -J. Am. Med. Assoc..

[98] Gao X.F., Ge Z., Kong X.-Q., Kan J., Han L., Lu S., Tian N.-L., Lin S., Lu Q.-H., Wang X.-Y. (2021). 3-Year Outcomes of the ULTIMATE Trial Comparing Intravascular Ultrasound Versus Angiography-Guided Drug-Eluting Stent Implantation. JACC Cardiovasc. Interv..

[99] Jia H., Dai J., He L., Xu Y., Shi Y., Zhao L., Sun Z., Liu Y., Weng Z., Feng X. (2022). EROSION III: A Multicenter RCT of OCT-Guided Reperfusion in STEMI with Early Infarct Artery Patency. JACC Cardiovasc. Interv..

[100] Gonzalo N., Barlis P., Serruys P.W., Garcia-Garcia H.M., Onuma Y., Ligthart J., Regar E. (2009). Incomplete Stent Apposition and Delayed Tissue Coverage Are More Frequent in Drug-Eluting Stents Implanted During Primary Percutaneous Coronary Intervention for ST-Segment Elevation Myocardial Infarction Than in Drug-Eluting Stents Implanted for Stable/Unstable Angina. Insights From Optical Coherence Tomography. JACC Cardiovasc. Interv..

[101] Van Geuns R.J., Tamburino C., Fajadet J., Vrolix M., Witzenbichler B., Eeckhout E., Spaulding C., Reczuch K., La Manna A., Spaargaren R. (2012). Self-expanding versus balloon-expandable stents in acute myocardial infarction: Results from the APPOSITION II Study: Self-expanding stents in ST-segment elevation myocardial infarction. JACC Cardiovasc. Interv..

[102] Leone A.M., Rebuzzi A.G., Burzotta F., De Maria G.L., Gardi A., Basile E., Cialdella P., D’Amario D., Paraggio L., Porto I. (2019). Stent malapposition, strut coverage and atherothrombotic prolapse after percutaneous coronary interventions in ST-segment elevation myocardial infarction. J. Cardiovasc. Med..

[103] Li X., Ge Z., Kan J., Anjum M., Xie P., Chen X., Khan H.S., Guo X., Saghir T., Chen J. (2024). Intravascular ultrasound-guided versus angiography-guided percutaneous coronary intervention in acute coronary syndromes (IVUS-ACS): A two-stage, multicentre, randomised trial. Lancet.

[104] Rissanen T.T., Uskela S., Eränen J., Mäntylä P., Olli A., Romppanen H., Siljander A., Pietilä M., Minkkinen M.J., Tervo J. (2019). Drug-coated balloon for treatment of de-novo coronary artery lesions in patients with high bleeding risk (DEBUT): A single-blind, randomised, non-inferiority trial. Lancet.

[105] Jeger R.V., Farah A., Ohlow M.-A., Mangner N., Möbius-Winkler S., Leibundgut G., Weilenmann D., Wöhrle J., Richter S., Schreiber M. (2018). Drug-coated balloons for small coronary artery disease (BASKET-SMALL 2): An open-label randomised non-inferiority trial. Lancet.

[106] Nijhoff F., Agostoni P., Belkacemi A., Nathoe H.M., Voskuil M., Samim M., Doevendans P.A., Stella P.R. (2015). Primary percutaneous coronary intervention by drug-eluting balloon angioplasty: The nonrandomized fourth arm of the DEB-AMI (drug-eluting balloon in ST-segment elevation myocardial infarction) trial. Catheter. Cardiovasc. Interv..

[107] Merinopoulos I., Gunawardena T., Corballis N., Bhalraam U., Reinhold J., Wickramarachchi U., Maart C., Gilbert T., Richardson P., Sulfi S. (2023). Assessment of Paclitaxel Drug-Coated Balloon Only Angioplasty in STEMI. JACC Cardiovasc. Interv..

[108] McNair P.W., Bilchick K.C., Keeley E.C. (2019). Very late presentation in ST elevation myocardial infarction: Predictors and long-term mortality. IJC Heart Vasc..

[109] Bouisset F., Gerbaud E., Bataille V., Coste P., Puymirat E., Belle L., Delmas C., Cayla G., Motreff P., Lemesle G. (2021). Percutaneous Myocardial Revascularization in Late-Presenting Patients with STEMI. J. Am. Coll. Cardiol..

[110] Schömig A., Mehilli J., Antoniucci D., Ndrepepa G., Markwardt C., Di Pede F., Nekolla S.G., Schlotterbeck K., Schühlen H., Pache J. (2005). Mechanical reperfusion in patients with acute myocardial infarction presenting more than 12 hours from symptom onset: A randomized controlled trial. JAMA.

[111] Lowe H.C., Freedman S.B. (2009). The late open artery hypothesis: The case and the artery remain closed. Eur. Heart J..

[112] Ioannidis J.P.A., Katritsis D.G. (2007). Percutaneous coronary intervention for late reperfusion after myocardial infarction in stable patients. Am. Heart J..

[113] Džavík V., Buller C.E., Lamas G.A., Rankin J.M., Mancini G.J., Cantor W.J., Carere R.J., Ross J.R., Atchison D., Thomas B. (2006). Randomized trial of percutaneous coronary intervention for subacute infarct-related coronary artery occlusion to achieve long-term patency and improve ventricular function: The Total Occlusion Study of Canada (TOSCA)-2 trial. Circulation.

[114] Busk M., Kaltoft A., Nielsen S.S., Bøttcher M., Rehling M., Thuesen L., Bøtker H.E., Lassen J.F., Christiansen E.H., Krusell L.R. (2009). Infarct size and myocardial salvage after primary angioplasty in patients presenting with symptoms for <12 h vs. 12-72 h. Eur. Heart J..

[115] Nepper-Christensen L., Lønborg J., Høfsten D.E., Ahtarovski K.A., Bang L.E., Helqvist S., Kyhl K., Køber L., Kelbæk H., Vejlstrup N. (2018). Benefit from reperfusion with primary percutaneous coronary intervention beyond 12 hours of symptom duration in patients with ST-segment-elevation myocardial infarction. Circ. Cardiovasc. Interv..

[116] Liu D., Borlotti A., Viliani D., Jerosch-Herold M., Alkhalil M., De Maria G.L., Fahrni G., Dawkins S., Wijesurendra R., Francis J. (2017). CMR Native T1 Mapping Allows Differentiation of Reversible Versus Irreversible Myocardial Damage in ST-Segment-Elevation Myocardial Infarction: An OxAMI Study (Oxford Acute Myocardial Infarction). Circ. Cardiovasc. Imaging.

[117] Dall’Armellina E., Piechnik S.K., Ferreira V.M., Le Si Q., Robson M.D., Francis J.M., Cuculi F., Kharbanda R.K., Banning A.P., Choudhury R.P. (2012). Cardiovascular magnetic resonance by non contrast T1-mapping allows assessment of severity of injury in acute myocardial infarction. J. Cardiovasc. Magn. Reson..

[118] De Maria G.L., Cuculi F., Patel N., Dawkins S., Fahrni G., Kassimis G., Choudhury R.P., Forfar J.C., Prendergast B.D., Channon K.M. (2015). How does coronary stent implantation impact on the status of the microcirculation during primary percutaneous coronary intervention in patients with ST-elevation myocardial infarction?. Eur. Heart J..

[119] De Waha S., Patel M.R., Granger C.B., Ohman E.M., Maehara A., Eitel I., Ben-Yehuda O., Jenkins P., Thiele H., Stone G.W. (2017). Relationship between microvascular obstruction and adverse events following primary percutaneous coronary intervention for ST-segment elevation myocardial infarction: An individual patient data pooled analysis from seven randomized trials. Eur. Heart J..

[120] Scarsini R., Shanmuganathan M., De Maria G.L., Borlotti A., Kotronias R.A., Burrage M.K., Terentes-Printzios D., Langrish J., Lucking A., Fahrni G. (2021). Coronary Microvascular Dysfunction Assessed by Pressure Wire and CMR After STEMI Predicts Long-Term Outcomes. JACC Cardiovasc. Imaging.

[121] Konijnenberg L.S.F., Damman P., Duncker D.J., Kloner R.A., Nijveldt R., van Geuns R.-J.M., Berry C., Riksen N.P., Escaned J., van Royen N. (2020). Pathophysiology and diagnosis of coronary microvascular dysfunction in ST-elevation myocardial infarction. Cardiovasc. Res..

[122] Niccoli G., Scalone G., Lerman A., Crea F. (2016). Coronary microvascular obstruction in acute myocardial infarction. Eur. Heart J..

[123] Van Kranenburg M., Magro M., Thiele H., de Waha S., Eitel I., Cochet A., Cottin Y., Atar D., Buser P., Wu E. (2014). Prognostic Value of Microvascular Obstruction and Infarct Size, as Measured by CMR in STEMI Patients. JACC Cardiovasc. Imaging.

[124] Carrick D., Haig C., Ahmed N., McEntegart M., Petrie M.C., Eteiba H., Hood S., Watkins S., Lindsay M.M., Davie A. (2016). Myocardial hemorrhage after acute reperfused ST-segment-elevation myocardial infarction: Relation to microvascular obstruction and prognostic significance. Circ. Cardiovasc. Imaging.

[125] Stone G.W., Selker H.P., Thiele H., Patel M.R., Udelson J.E., Ohman E.M., Maehara A., Eitel I., Granger C.B., Jenkins P.L. (2016). Relationship Between Infarct Size and Outcomes Following Primary PCI Patient-Level Analysis From 10 Randomized Trials. J. Am. Coll. Cardiol..

[126] Fearon W.F., Balsam L.B., Farouque H.M.O., Robbins R.C., Fitzgerald P.J., Yock P.G., Yeung A.C. (2003). Novel index for invasively assessing the coronary microcirculation. Circulation.

[127] Heusch G., Bøtker H.E., Przyklenk K., Redington A., Yellon D. (2015). Remote ischemic conditioning. J. Am. Coll. Cardiol..

[128] El Farissi M., Zimmermann F.M., De Maria G.L., van Royen N., Vanleeuwen M.A.H., Carrick D., Carberry J., Wijnbergen I.F., Konijnenberg L.S.F., Hoole S.P. (2023). The Index of Microcirculatory Resistance After Primary PCI: A Pooled Analysis of Individual Patient Data. JACC Cardiovasc. Interv..

[129] Fearon W.F., Low A.F., Yong A.S., McGeoch R., Berry C., Shah M.G., Ho M.Y., Kim H.-S., Loh J.P., Oldroyd K.G. (2013). Prognostic value of the Index of Microcirculatory Resistance measured after primary percutaneous coronary intervention. Circulation.

[130] De Maria G.L., Alkhalil M., Wolfrum M., Fahrni G., Borlotti A., Gaughran L., Dawkins S., Langrish J.P., Lucking A.J., Choudhury R.P. (2019). Index of Microcirculatory Resistance as a Tool to Characterize Microvascular Obstruction and to Predict Infarct Size Regression in Patients with STEMI Undergoing Primary PCI. JACC Cardiovasc. Imaging.

[131] De Maria G.L., Fahrni G., Alkhalil M., Cuculi F., Dawkins S., Wolfrum M., Choudhury R., Forfar J., Prendergast B., Yetgin T. (2016). A tool for predicting the outcome of reperfusion in ST-elevation myocardial infarction using age, thrombotic burden and index of microcirculatory resistance (ATI score). EuroIntervention.

[132] De Maria G.L., Alkhalil M., Wolfrum M., Fahrni G., Borlotti A., Gaughran L., Dawkins S., Langrish J., Lucking A., Choudhury R. (2017). The ATI score (age-thrombus burden-index of microcirculatory resistance) determined during primary percutaneous coronary intervention predicts final infarct size in patients with ST-elevation myocardial infarction: A cardiac magnetic resonance validation study. EuroIntervention.

[133] Fahrni G., Wolfrum M., De Maria G.L., Cuculi F., Dawkins S., Alkhalil M., Patel N., Forfar J.C., Prendergast B.D., Choudhury R.P. (2017). Index of microcirculatory resistance at the time of primary percutaneous coronary intervention predicts early cardiac complications: Insights from the OxAMI (Oxford Study in Acute Myocardial Infarction) Cohort. J. Am. Heart Assoc..

[134] Scarsini R., Portolan L., Della Mora F., Marin F., Mainardi A., Ruzzarin A., Levine M.B., Banning A.P., Ribichini F., Garcia H.M.G. (2023). Angiography-Derived and Sensor-Wire Methods to Assess Coronary Microvascular Dysfunction in Patients with Acute Myocardial Infarction. JACC Cardiovasc. Imaging.

[135] Kotronias R.A., Fielding K., Greenhalgh C., Lee R., Alkhalil M., Marin F., Emfietzoglou M., Banning A.P., Vallance C., Channon K.M. (2022). Machine learning assisted reflectance spectral characterisation of coronary thrombi correlates with microvascular injury in patients with ST-segment elevation acute coronary syndrome. Front. Cardiovasc. Med..

[136] Scarsini R., Kotronias R.A., Della Mora F., Portolan L., Andreaggi S., Benenati S., Marin F., Sgreva S., Comuzzi A., Butturini C. (2024). Angiography-Derived Index of Microcirculatory Resistance to Define the Risk of Early Discharge in STEMI. Circ. Cardiovasc. Interv..

[137] Bartorelli A.L. (2003). Hyperoxemic Perfusion for Treatment of Reperfusion Microvascular Ischemia in Patients with Myocardial Infarction. Am. J. Cardiovasc. Drugs.

[138] Watanabe S., Fish K., Kovacic J.C., Bikou O., Leonardson L., Nomoto K., Aguero J., Kapur N.K., Hajjar R.J., Ishikawa K. (2018). Left ventricular unloading using an impella CP improves coronary flow and infarct zone perfusion in ischemic heart failure. J. Am. Heart Assoc..

[139] Esposito M.L., Zhang Y., Qiao X., Reyelt L., Paruchuri V., Schnitzler G.R., Morine K.J., Annamalai S.K., Bogins C., Natov P.S. (2018). Left Ventricular Unloading Before Reperfusion Promotes Functional Recovery After Acute Myocardial Infarction. J. Am. Coll. Cardiol..

[140] Kapur N.K., Alkhouli M., DeMartini T.J., Faraz H., George Z.H., Goodwin M.J., Hernandez-Montfort J.A., Iyer V.S., Josephy N., Kalra S. (2019). Unloading the Left Ventricle Before Reperfusion in Patients with Anterior ST-Segment-Elevation Myocardial Infarction: A Pilot Study Using the Impella CP. Circulation.

[141] Kapur N.K., Kim R.J., Moses J.W., Stone G.W., Udelson J.E., Ben-Yehuda O., Redfors B., Issever M.O., Josephy N., Polak S.J. (2022). Primary left ventricular unloading with delayed reperfusion in patients with anterior ST-elevation myocardial infarction: Rationale and design of the STEMI-DTU randomized pivotal trial. Am. Heart J..

[142] Svilaas T., Van Der Horst I.C.C., Zijlstra F. (2006). Thrombus Aspiration during Percutaneous coronary intervention in Acute myocardial infarction Study (TAPAS)—Study design. Am. Heart J..

[143] Lagerqvist B., Fröbert O., Olivecrona G.K., Gudnason T., Maeng M., Alström P., Andersson J., Calais F., Carlsson J., Collste O. (2014). Outcomes 1 Year after Thrombus Aspiration for Myocardial Infarction. N. Engl. J. Med..

[144] Jolly S.S., Cairns J.A., Yusuf S., Rokoss M.J., Gao P., Meeks B., Kedev S., Stankovic G., Moreno R., Gershlick A. (2016). Outcomes after thrombus aspiration for ST elevation myocardial infarction: 1-year follow-up of the prospective randomised TOTAL trial. Lancet.

[145] Bhindi R., Kajander O.A., Jolly S.S., Kassam S., Lavi S., Niemelä K., Fung A., Cheema A.N., Meeks B., Alexopoulos D. (2015). Culprit lesion thrombus burden after manual thrombectomy or percutaneous coronary intervention-alone in ST-segment elevation myocardial infarction: The optical coherence tomography sub-study of the TOTAL (ThrOmbecTomy versus PCI ALone) trial. Eur. Heart J..

[146] Higuma T., Soeda T., Yamada M., Yokota T., Yokoyama H., Izumiyama K., Nishizaki F., Minami Y., Xing L., Yamamoto E. (2016). Does Residual Thrombus After Aspiration Thrombectomy Affect the Outcome of Primary PCI in Patients with ST-Segment Elevation Myocardial Infarction? An Optical Coherence Tomography Study. Cardiovasc. Interv..

[147] Berkhemer O., Fransen P.S.S., Beumer D., Berg L.A.V.D., Lingsma H.F., Yoo A.J., Schonewille W.J., Vos J.A., Nederkoorn P.J., Wermer M.J.H. (2015). A Randomized Trial of Intraarterial Treatment for Acute Ischemic Stroke. N. Engl. J. Med..

[148] Mathews S.J., Parikh S.A., Wu W., Metzger D.C., Chambers J.W., Ghali M.G., Sumners M.J., Kolski B.C., Pinto D.S., Dohad S. (2023). Sustained Mechanical Aspiration Thrombectomy for High Thrombus Burden Coronary Vessel Occlusion: The Multicenter CHEETAH Study. Circ. Cardiovasc. Interv..

[149] Ciofani J.L., Allahwala U.K., Scarsini R., Ekmejian A., Banning A.P., Bhindi R., De Maria G.L. (2021). No-reflow phenomenon in ST-segment elevation myocardial infarction: Still the Achilles’ heel of the interventionalist. Future Cardiol..

[150] McCartney P.J., Eteiba H., Maznyczka A.M., McEntegart M., Greenwood J.P., Muir D.F., Chowdhary S., Gershlick A.H., Appleby C., Cotton J.M. (2019). Effect of Low-Dose Intracoronary Alteplase during Primary Percutaneous Coronary Intervention on Microvascular Obstruction in Patients with Acute Myocardial Infarction: A Randomized Clinical Trial. JAMA-J. Am. Med. Assoc..

[151] Niccoli G., Rigattieri S., De Vita M.R., Valgimigli M., Corvo P., Fabbiocchi F., Romagnoli E., De Caterina A.R., La Torre G., Lo Schiavo P. (2013). Open-label, randomized, placebo-controlled evaluation of intracoronary adenosine or nitroprusside after thrombus aspiration during primary percutaneous coronary intervention for the prevention of microvascular obstruction in acute myocardial infarction: The REOPEN-AMI study (Intracoronary Nitroprusside Versus Adenosine in Acute Myocardial Infarction). JACC Cardiovasc. Interv..

